# Mass Purification Protocol for *Drosophila melanogaster* Wing Imaginal Discs: An Alternative to Dissection to Obtain Large Numbers of Disc Cells

**DOI:** 10.3390/biology11101384

**Published:** 2022-09-22

**Authors:** Marion Hoareau, Juliette de Noiron, Jessie Colin, Isabelle Guénal

**Affiliations:** 1 LGBC, UVSQ, Université Paris-Saclay, 78000 Versailles, France; 2 Ecole Pratique des Hautes Etudes, PSL Research University, 75014 Paris, France

**Keywords:** *Drosophila melanogaster*, larva, mass purification, isolation, wing imaginal discs, density gradient

## Abstract

**Simple Summary:**

*Drosophila melanogaster*, also known as the fruit fly, is a widely used organism model, especially for genetic studies or as a model for pathologies. The *Drosophila* genome is well known and conserved within humans, thus allowing biologists to obtain numerous mutants and transgenic flies from the fruit flies. Gene function studies at the cellular and molecular levels are often performed using extracts of larval tissues. Due to their small size, it is difficult to dissect substantial amounts of these tissues for performing genomic or proteomic experiments. This paper develops a simple method to purify larval tissues en masse. This protocol preserves tissue integrity in the same way as manual dissection; the protocol is achievable by individual researchers and allows the purification of different samples simultaneously.

**Abstract:**

*Drosophila melanogaster* imaginal discs are larval internal structures that become the external organs of the adult. They have been used to study numerous developmental processes for more than fifty years. Dissecting these imaginal discs for collection is challenging, as the size of third-instar larvae organs is typically less than 1 mm. Certain experimental applications of the organs require many cells, which requires researchers to spend several hours dissecting them. This paper proposes an alternative to dissection in the form of a mass enrichment protocol. The protocol enables the recovery of many wing imaginal discs by grinding large quantities of third-instar larvae and separating the organs using filtration and a density gradient. The wing imaginal discs collected with this protocol in less than three hours are as well preserved as those collected by dissection. The dissociation and filtration of the extract allow the isolation of a large amount of wing imaginal disc cells.

## 1. Introduction

In Holometabola, imaginal discs are larval internal epithelial structures that are precursors of external organs in the adult, such as wings or legs. These structures are widely used in the model organism *Drosophila melanogaster* for developmental studies. The structures are accessible, and most genetic tools engineered for this species are designed to be used in these organs, including drivers to use the UAS-GAL4 system [1] or mitotic clones [2] enabling constructs, among others. Many genes and signaling cascades involved in developmental control are conserved between mammals and *Drosophila melanogaster* [3], making imaginal discs an interesting model to study processes involved in both development [4,5] and human diseases (see for example [6,7]). Such studies led to significant discoveries in recent decades, including homeotic genes [8] and the Hippo pathway [9]. However, although *Drosophila* wing imaginal disc has proven to be a great model for genetic or cell biology approaches, its use is more difficult in biochemistry or molecular biology experiments that need large amounts of starting material. For instance, a chromatin immunoprecipitation (ChIP) requires 400 wing imaginal discs per sample [10], and this number can be even higher if the factor of interest has a low concentration or is expressed only in a subpopulation of the disc cells. Manual dissection is the classical way to collect imaginal discs. Thus, doing experiments such as a ChIP with associated corresponding controls rapidly leads to the need for dissecting hundreds of larvae. Dissection is time-consuming and raises reproducibility issues, as several scientists may be involved. Subtle variations may occur throughout the dissection process, imaginal disc storage, or crosslinking step. Molecular biology experiments thus necessitate an alternative method to isolate imaginal discs en masse, in a reproducible manner, and by a single experimenter in a limited amount of time.

Since imaginal discs such as wing ones are precursors of non-essential organs, they are instrumental in studying cell death as it is possible to induce cell death during the development in these structures without affecting the survival of the specimen [11]. Our team is interested in apoptosis and tissue homeostasis. Our favorite model organ is the wing imaginal disc [12,13]. However, as our projects necessitated a large number of discs, we decided to invest in developing a mass wing imaginal discs enrichment protocol. This protocol would allow us to harvest a significantly greater sample size of wing imaginal discs than previously achieved for larger-scale experiments. A few articles describe such protocols. The first two were published in the 1960 and 1970s [14,15] and offer mass isolation methods that allow the recovery of hundreds of imaginal discs using density gradients. Methods related to the large-scale collection of fruit fly larval tissues described during the 1960–1970s [14,15] cannot be easily reproduced due to the lack of documentation of methods and the unavailability of some tools. More recently, Marty et al. [16] presented a new version of this protocol, but their goal was only to roughly separate organs and not to obtain pure imaginal disc fractions. In this study, the authors used a Biosorter^®^ (Union Biometrica Inc., Holliston, MA, USA) to isolate wing imaginal discs according to their shape, size, or Green Fluorescent Protein (GFP) pattern. However, this device is expensive and not commonly available. Therefore, we wanted to develop a mass enrichment protocol routinely usable in any laboratory.

**Figure 1:**
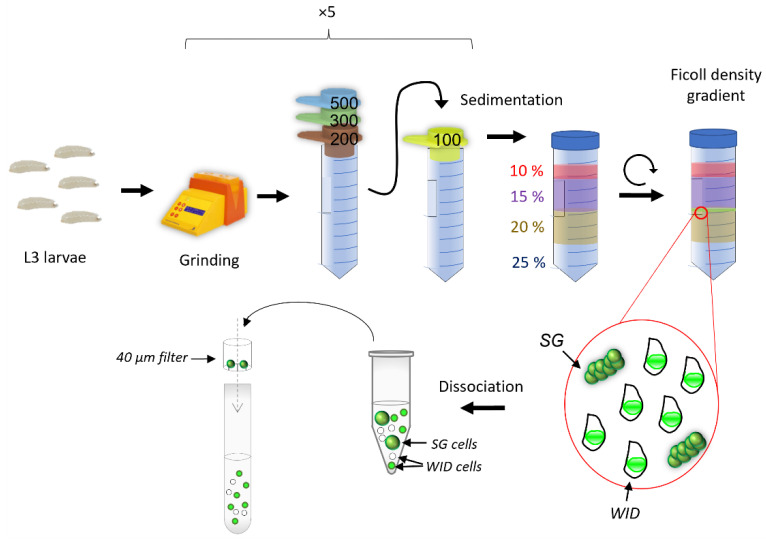
Overview of the mass purification protocol. L3 larvae obtained from synchronized egg-laying are collected en masse using a wash bottle. The larvae were initially ground using a GentleMACS^TM^ (Miltenyi Biotec, Bergisch Gladbach, North Rhine-Westphalia, Germany). The material was then filtered through a series of strainers with decreasing mesh size, allowing the selection of elements between 100 µm and 200 µm. This material is resuspended in 10% (*w*/*v*) Ficoll and loaded on top of a 15:20:25% (*w*/*v*) Ficoll gradient. After centrifugation, wing imaginal discs (WID) are found at the 15:20% (*w*/*v*) interface along with some salivary gland (SG) pieces. After dissociation, filtering the cells through a 40 µm filter is enough to separate salivary gland cells from disc cells.

We propose here an imaginal disc mass enrichment protocol without manual dissection that allows the recovery of a large number of discs in an optimized time range, doable entirely by one experimenter, using density gradients and sedimentation; see the overall protocol in Figure 1. This method allows the experimenter to recover about 11.5% of the wing imaginal discs input within two to three hours in an enriched fraction (about 50%) that can be easily dissociated and filtrated to recover the wing imaginal disc cells. The protocol is optimized for wing imaginal discs, but its adaptation to other larvae organs such as eye-antenna imaginal discs or brains could be possible after different set-ups, especially the nature of the Ficoll gradient layers.

## 2. Materials and Methods

### 2.1. Fly Stocks

Flies were raised at 25 °C on a standard medium. The *vg-GAL4* strain is a generous gift from Joel Silber (Institut Jacques Monod, Université Paris Cité, France). The *UAS-mCD8-GFP* was obtained from the Bloomington Drosophila Stock Center (BL-32185).

### 2.2. Protocol for Mass Enrichment of Wing Imaginal Discs

Third-instar larvae were collected by flushing the side of their rearing tubes. The larvae were then ground using the GentleMACS™ device (Miltenyi Biotec, Bergisch Gladbach, Germany) and filtered through a series of sieves. The resulting material was loaded in 10% Ficoll solution (*w*/*v*) on top of a 15:20:25% gradient. After centrifugation, the 15:20% interface containing the enriched wing imaginal discs was collected and rehydrated in Ringer 1X (Supplementary File S1). After dissociation, filtration on 40 µm retained the cells of the salivary glands and allowed the recovery of only imaginal disc cells.

### 2.3. Immunostaining and Images Acquisition

For the “dissected” condition, wing imaginal discs were dissected from third-instar larvae in 1X Phosphate Buffered Saline (PBS), pH 7.6. Discs obtained by mass enrichment or dissection were fixed with 3.7% formaldehyde in 1X PBS for 20 min at room temperature and washed three times for 10 min in PBST (1X PBS, 0.3% Triton X-100). 

Apoptosis was analyzed as previously described in de Noiron et al. [17]. In brief, the discs were blocked for 1 h in PBST-BSA (1X PBS, 0.3% Tween 20, 2% Bovin Serum Albumin) and incubated overnight with 1:100 dilution of anti-cleaved *Drosophila* Dcp-1 (Asp216, Cell Signaling Technology) at 4 °C. The following day, after 3 washes in PBST, wing discs were incubated for two hours with Alexa-568-coupled anti-rabbit secondary antibody (A-11011, Invitrogen) diluted to 1:400 in PBST. Finally, wing discs were mounted in ProLong Diamond (Invitrogen, Waltham, MA, USA), and images were acquired using a Leica SP8 confocal microscope (Leica Camera, Wetzlar, Germany) at 568 nm. At least 30 wing imaginal discs are analyzed for each condition. Image analysis was performed on Fiji with the macro described in de Noiron_2021 [17], whose main steps are median filter application, stacking (Z-project), threshold determination, and signal quantification.

For tissue structure analysis, wing imaginal discs were incubated with phalloidin-ATTO 665 (Sigma-Aldrich, St. Louis, MO, USA) 1 μg/mL, and Hoechst 33,342 (Thermo Fisher Scientific, Waltham, MA, USA) 1 μg/mL, in PBST-BSA for 1 h at room temperature. Larvae were then washed twice for 5 min in PBST. Finally, wing discs were mounted in Citifluor^TM^ (Biovalley, Basel, Switzerland) and observed with a Leica SP8 upright confocal microscope.

### 2.4. Flow Cytometry Analysis

Third-instar larvae expressing *UAS-mCD8-GFP* under the control of the *vestigial-GAL4* driver were used in [18]. Wing imaginal discs recovered by either dissection or mass enrichment protocol were dissociated using a 1:50 dilution (final concentration 0.1% *w*/*v*) of 5% (*w*/*v*)% protease (Sigma-Aldrich P8811) in 1X Ringer incubated for 20 min at 25 °C under gentle shaking (300 rpm). Dissociation was completed by gently passing the cells through a P1000 tip. After pelleting, cells were resuspended in 1X Ringer and filtered through a 40 µm sieve to retain salivary glands cells and eventual debris. Cells were then analyzed using the BD FACSAria™ III (BD Biosciences, Franklin Lakes, NJ, USA) equipped with a 488 nm laser line and the BD FACSDiva™ software (9.0.1, BD Biosciences, Franklin Lakes, NJ, USA). Cells were selected through forward scatter (FSC) to keep out debris and cell clusters. They were then sorted based on whether or not they expressed GFP.

For the cell death assessment by viability dye, 10 third-instar larvae expressing *UAS-mCD8-GFP* under the control of the *vestigial-GAL4* driver were used for each group. Wing imaginal discs recovered by either dissection in 1X PBS, dissection in 1X Ringer, or mass enrichment protocol in 1X Ringer were dissociated using a 0.1% (*w*/*v*) solution of protease (Sigma-Aldrich P8811) in a 1X PBS or 1X Ringer and incubated for 20 min at 25 °C under gentle shaking (300 rpm). Dissociation was completed by gently passing the cells through a P1000 tip. After pelleting, the cells were incubated for 3 min on ice in viability dye solution (Fixable Viability Dye eFluor™ 780, eBioscience™, Thermo Fisher Scientific, Waltham, MA, USA) diluted to 1:1000 in 1X PBS or 1X Ringer. After a wash in 1X PBS or 1X Ringer, cells were resuspended in 1X PBS or 1X Ringer and filtered through a 40 µm sieve. The fluorescence of the dye was then measured using the BD FACSARia™ III equipped with a 635 nm laser line and the BD FACSDiva™ software. Cells were selected through FSC, excluding debris and cell clusters. Data analysis was performed with FlowJo software (10.8.1 version, FlowJo, Ashland, OR, USA). Graphs and statistical analysis were performed using RStudio (ggplot2 and ggpubr packages, RStudio, Boston, MA, USA) with a significance threshold of 5%.

## 3. Results

### 3.1. Grinding and Buffer Parameters to Release Internal Organs without Damaging Them

Few protocols of mass enrichment for imaginal discs have previously been described. They are all based on the protocol developed by Fristrom and Mitchell [14], but their use needs some modernizing. This protocol requires dismantling larvae to release internal organs later isolated by differential sedimentation. As we mainly work on wing imaginal discs, we focused on this organ and chose to monitor its enrichment process with the help of fluorescent wing discs. To this end, we used larvae expressing GFP under the control of the vestigial driver, a driver allowing expression in a wide band at the dorso-ventral frontier of wing and haltere wing discs. Like many other wing imaginal disc drivers [1,19], it also displays leaky expression in the salivary glands, which is not an issue as salivary glands are easily distinguishable from wing imaginal discs.

#### 3.1.1. Grinding Larvae: A Fine-Tuning between Breaking the Cuticle and Keeping the Organs Undamaged

The first step of this protocol consists of dismantling the larvae without damaging the imaginal discs. Protocols from Mitchell and Cohen laboratories used either a meat grinder that is in all likelihood not sold anymore [14] or a custom-made device specifically developed in this laboratory for this particular usage [15]. Our goal was to use a commercial tissue dissociator to ensure the reproducibility of the larvae grinding. Thus, we tried two devices. The first was a bead beater like the ones usually available in most laboratories, in our case, the Precellys^®^ (Bertin Technologies, Montigny-le-Bretonneux, France) from Bertin. The second was the GentleMACS™ from Miltenyi, which was successfully used for such applications by the Basler lab [16]. We expected it to perform a gentle grinding, as it was developed for tissue dissociation rather than whole lysis. As these devices are usually dedicated to mammalian tissue lysis or dissociation, their ability to grind *Drosophila* larvae is not documented. The grinding process was visually monitored by assessing the integrity and location (released from the larvae or not) of fluorescent discs at the end of the process.

The development tests were mainly carried out using 2.5 mL of larvae, representing around 600 individuals. This larvae amount is collected by flushing the side of six vials. Adult flies were transferred to a fresh medium every 24 h to ensure that all offspring were the same age and obtained in sufficient quantity. We used 12 vials containing 15 virgin females and 6 males to collect the same amount (2.5 mL) of larvae from a cross where the larvae of interest represented only 50% of the progeny. These values must be adapted according to the viability of the individuals of interest.

For the assays with the Precellys^®^, we used 2 mL tubes containing either 2.8 mm diameter beads or a mix of 1.4 and 2.8 mm beads with different grinding cycles. We found that 2.8 mm beads alone were more efficient in breaking cuticles and that the recovered imaginal discs were in better condition than using the bead mix. However, the yield was low since most discs remained attached to the larvae (data not shown). Furthermore, considering the quick mass enrichment protocol we wanted to set up, we did not further pursue the set-up with this device as it can only handle a limited number of larvae (100–120/tube) at once, and the recovery of the material after grinding was challenging to handle—these drawbacks are incompatible with mass enrichment.

The GentleMACS™ uses tubes with spinning helix-like structures and comes with several pre-registered programs of different speeds and/or duration. Two types of tubes are available; the C tubes offer a gentler grinding and are dedicated to tissue dissociation into individual cells, whereas M tubes offer stronger grinding and are recommended for complete cell lysis as required for biomolecule extraction. We first used the “liver 1” programs (48 rotations per round, 15 s) that were previously used for such purpose [16] or “liver 2” programs (78 rpr, 24 s) with the two kinds of tubes (Supplementary Figure S1). In our hands, these programs were too gentle to detach the imaginal discs efficiently, and upon advice from the technical support from Miltenyi Biotec, we tested the “brain1” programs (116 rpr, 36 s) or “brain” 2 programs (100 rpr, 30 s), the second of which turned out to be much more appropriate. We observed that with the M tubes, larvae were ground either not enough or too much, resulting in damaged imaginal discs. By contrast, the C tubes allowed a dismantling of the larvae that released intact imaginal discs (Supplementary Figure S1). We used the GentleMACS™ with the “brain 2” program and the C tubes based on these tests. This grinding step is repeated five times until most, if not all, internal organs are released from the larvae.

#### 3.1.2. Filtration and Ficoll Gradient: Steps to Separate Organs of Interest from the Others

**Figure 2:**
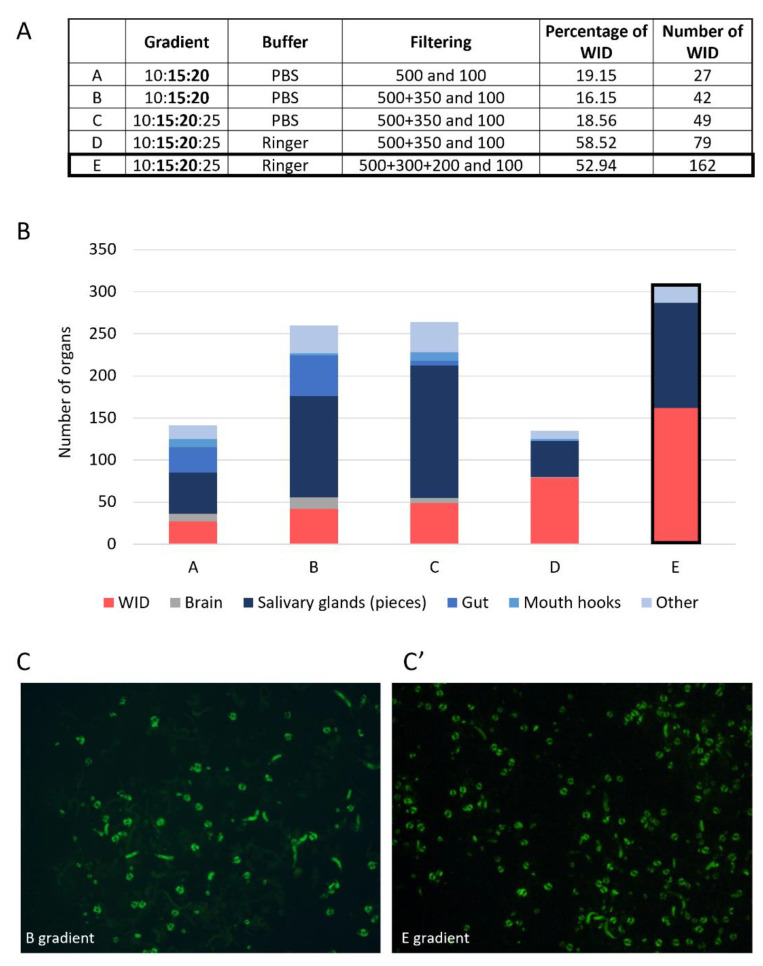
Overview of the improvement in the yield and purity during Ficoll gradient set-up. The “E” gradient is used in this protocol final version. (**A**) Table showing 5 examples of gradient configuration (Ficoll gradient in %, filtering in µm (pore size)) and their outcome in proportion and number of wing imaginal discs (WID). Inputs were 5 mL of larvae. (**B**) Count of the different organs obtained in the output fractions. The “Other” group includes other imaginal discs (mainly legs and eye-antennae), proventriculus, and pieces of cuticles. (**C**) Examples of fractions obtained with the B (**C**) and E (**C’**) gradients in GFP-exciting light, ×18. All pseudocircular GFP-marked objects are wing imaginal discs with *vg*-driven GFP expression.

The second step of the protocol consists of isolating imaginal discs from the other organs. As per the protocol from Marty [16], we first loaded the ground material, without further treatment, directly on a Ficoll gradient. However, this was not satisfying, since it led to a gradient saturation. We then added a step of rough separation of the organs based on their size. To this end, we used a series of filters with different meshing. We first tried a simple filtration step with only a 100 µm strainer that kept wing imaginal discs and let us eliminate little pieces of organs (such as gut pieces). However, the ground fractions also contain many contaminants of larger size and/or density, such as pieces of cuticles or mouth hooks. Given the amount of material loaded on the filter, it was still rapidly clogged with these tissues. We thus decided to add a 500 µm filter before the 100 µm one to eliminate large structures, such as cuticles or tiny unground larvae, but the output of this 500 µm was still not clean enough. After numerous tries with different strainer sizes, we combined of 500 µm, 300 µm, and 200 µm in serial filtration (Figure 2). This filter combination retains all empty cuticles, larvae that are not fully grinded, and most mouth hooks while letting imaginal discs go through. This filtrate then undergoes 100 µm filtration that retains imaginal discs but allows small-sized contaminants such as fat body and small gut pieces to be removed. The material recovered from the 100 µm strainer contains imaginal discs and structures with the same size range, such as brains, proventriculus, “string-like” structures such as salivary glands and gut pieces, and some fat body pieces.

Still, to avoid gradient saturation, we added a sedimentation step. Indeed, this was a means to further improve the purity as gut and fat body pieces are much less dense than wing imaginal discs. To assess the efficiency of the sedimentation and purification steps, we needed a way to visually localize wing imaginal discs in the tubes. To do so, we took advantage of LacZ expression in those discs and added a fixation-less staining step of the beta-galactosidase [20] (Supplementary Figure S2). Finally, sedimentation with low-speed centrifugation allowed cleaning out this fraction. 

Once the input for the Ficoll gradient separation was cleared from most unwanted larval elements, we worked on the enrichment step. We tried many gradient configurations (number, concentration, and volume of layers). We present here only a subset of them representing the milestones of the development of the method (Figure 2). The most recently published protocol uses a Ficoll gradient prepared with PBS, which allows the recovery of the imaginal discs at the 16:25% interface [16]. The oldest method comprises two gradients, the first placing imaginal discs at the interface with 14:19% of a Ringer-prepared Ficoll gradient and the second allowing further separation of this fraction using a continuous 14–24% gradient [14]. The latest should provide the purest imaginal discs fractions, whereas the most recent gives much less pure preparation, but this was not a problem for the authors since they further sorted the enriched discs using a BioSorter. We wanted our method to stand in between, being the easiest and shortest possible but still favoring purity over yield. We then decided to improve the quality of the output fraction using a single gradient. We started with PBS-prepared Ficoll gradients, as used in the single gradient protocol. As in previous experiments, discs were found in the interphase in the 14–25% range. We expected a disc enrichment in the 15:20% interface, with 15 and 20% layers becoming the basic set-up of our method. We soon noticed that adding a layer with more diluted Ficoll (i.e., 10%) improved the separation. Adding another layer of 25% Ficoll at the bottom of the gradient further improved the enrichment of imaginal discs. Our final protocol corresponds to the “E” gradient in Figure 2A. It comprises a Ficoll gradient with a first 10% layer in which the ground material is resuspended. This layer is then loaded on top of a 15:20:25% gradient. Using filter tips coated with fat larvae tissue prevents the loss of wing imaginal discs sticking to the tips. This improvement and precise pipetting at the interphase of the gradient significantly increased the protocol yield compared to the one in Figure 2A “E”. Indeed, in six enrichment experiments from 2.5 mL of third instar larvae (about 600 larvae), we obtained an average of 138 wing imaginal discs per preparation, corresponding to a yield of 11.5%.

After centrifugation, wing imaginal discs, along with eye and antenna discs and salivary gland pieces, were found at the 15:20% interface. Some more wing imaginal discs could be recovered at the 20:25% interface, but we chose not to recover them because the interface also contains the vast majority of salivary glands and mouth hooks. For our applications, cells from imaginal discs were easy to separate from salivary gland cells, which are the primary contaminants in this case.

#### 3.1.3. Finding the Best Buffer for Isolating and Maintaining Intact Imaginal Discs

An important difference between the protocols of Marty et al. 2014 [16] and Fristrom and Mitchell 1965 [14] is the buffer used throughout the enrichment process. The original article by Fristrom and Mitchell [14] used a Ringer solution [21], whereas the most recently used solution was PBS. We started setting up the protocol using PBS, the cheapest and most commonly used buffer. However, after Ficoll gradient enrichment, the recovered discs looked dehydrated (data not shown). Since discs recovered in Fristrom and Mitchell’s article [14], for which Ringer solution was used, could be successfully transplanted, we considered switching to this buffer. After replacing PBS with Ringer solution during manual dissections, we observed that discs had a better appearance. We performed a viability assay using a dye that enters damaged cells with a permeabilized membrane to assess whether imaginal disc cells were indeed in more physiological conditions in a Ringer solution than in PBS.

**Figure 3:**
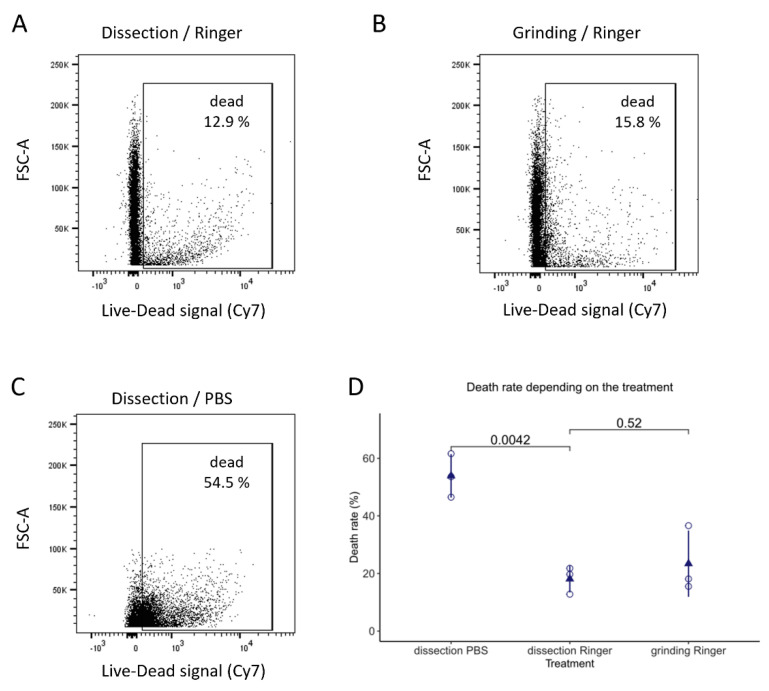
Effects of buffer and disc recovery method on cell viability. Wing imaginal discs obtained by either dissection (**A**,**C**) or grinding (**B**) in 1X Ringer (**A**,**B**) or 1X PBS (**C**) were dissociated, filtered through a 40 μm sieve, and stained with a viability dye (Fixable Viability Dye eFluor™ 780) that enters damaged cells with a permeabilized membrane. The dye intensity was measured by flow cytometry (635 nm) to assess the death rate (expressed in % of the total number of individual cells). (**A**–**C**) Cytograms correspond to FSC = cell size versus Live-Dead signal (Cy7). (**D**) The experiment was performed three times, and each point on the dot plot represents the death rate for one experiment. The triangular point represents the mean for each data set. Statistical significance was determined by one-way ANOVA using Bonferroni’s correction for multiple comparison testing.

We used this assay to determine whether one of the buffers, PBS or Ringer, is more damaging than the other. Confirming our observation, PBS is a much more damaging agent as cells obtained by dissection in PBS were more than twice as frequently labeled compared to cells treated in Ringer solution (Figure 3D). In addition, it could be envisioned that the mass enrichment process may be more damaging than dissection. Grinding could exert more mechanical stress than dissection, while using the Ficoll polymer may induce osmotic stress. The analysis of the impact of the recovery method showed that the mass enrichment process is not more damaging for the cells than dissection in this assay (Figure 3, compare A and B and see D). To further ensure that the mass enrichment process did not induce ectopic apoptosis, we performed apoptotic cell labeling using an antibody against activated caspases (anti-cleaved Dcp-1) [22]. This staining showed that mass enrichment does not induce more apoptosis than classical manual dissection (Supplementary Figure S3). The slight difference between those two conditions could be explained by the difference in the buffer used (PBS for dissection, Ringer for grinding). Finally, switching PBS to the Ringer solution improved the quality of the material recovered and the enrichment process (compare Figure 2A protocol “C” with Figure 2A protocol “D” and “E”) as it decreased the number of contaminants while increasing the number of discs recovered. 

**Figure 4:**
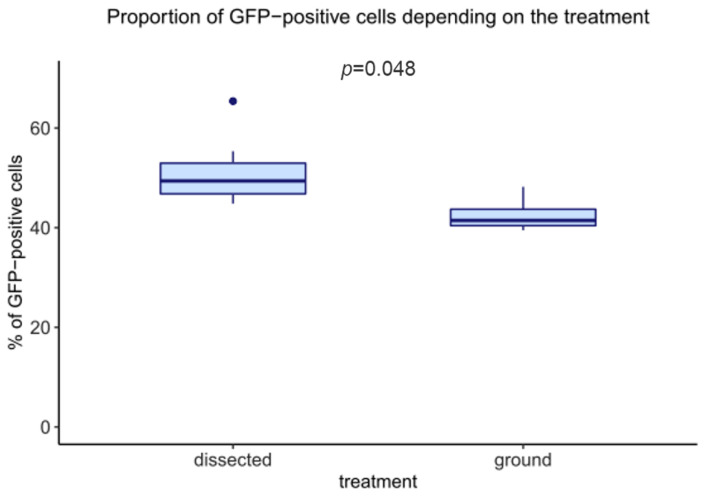
Proportion of GFP-positive cells in wing imaginal discs recovered by mass-enrichment protocol or dissection. *vg* > GFP expressing wing discs were recovered by either dissection or using our mass enrichment protocol, were dissociated, and the number of GFP-positive cells was measured by flow cytometry. A Wilcoxon test was performed for comparison (*n* = 12).

We continued the quality control of the material recovered by mass enrichment by analyzing the number of GFP-positive cells using flow cytometry. This analysis was carried out on larvae expressing the GFP in the wing disc under the control of the *vg-GAL4* driver. This driver induces GFP expression in a wide band at the dorso-ventral frontier, representing roughly 40% of the cells. Visual inspection of the GFP pattern readily allows the recognition of the *vg* pattern, confirming that discs do not undergo such stress that would lead to GFP extinction. Since salivary gland cells are bigger than wing imaginal disc cells [23], it is easy to discard them; after dissociation, passing the cell suspension through a 40 µm filter is enough to retain the bigger cells. After dissociation, filtration, and flow cytometry analysis of either mass-enriched or dissected wing imaginal discs, the percentage of GFP-positive cells was measured (Figure 4). 

Mass enrichment of discs led to a slight decrease in the proportion of GFP-positive cells compared with dissected discs (from 47.7% to 42.7%), but this decrease was expected as ground samples were not 100% pure. This fraction contains other organs (such as other discs) whose cells do not express GFP, thus increasing the GFP-negative fraction. This limited decrease indicates that the wing imaginal disc fraction recovered by mass enrichment is relatively pure. This result confirms our visual assessment and is supported by the fact that after dissociation and filtration, whatever the protocol used, the percentage of cells recovered compared to the total number of events counted by cytometry is not significantly different (Figure 4).

**Figure 5:**
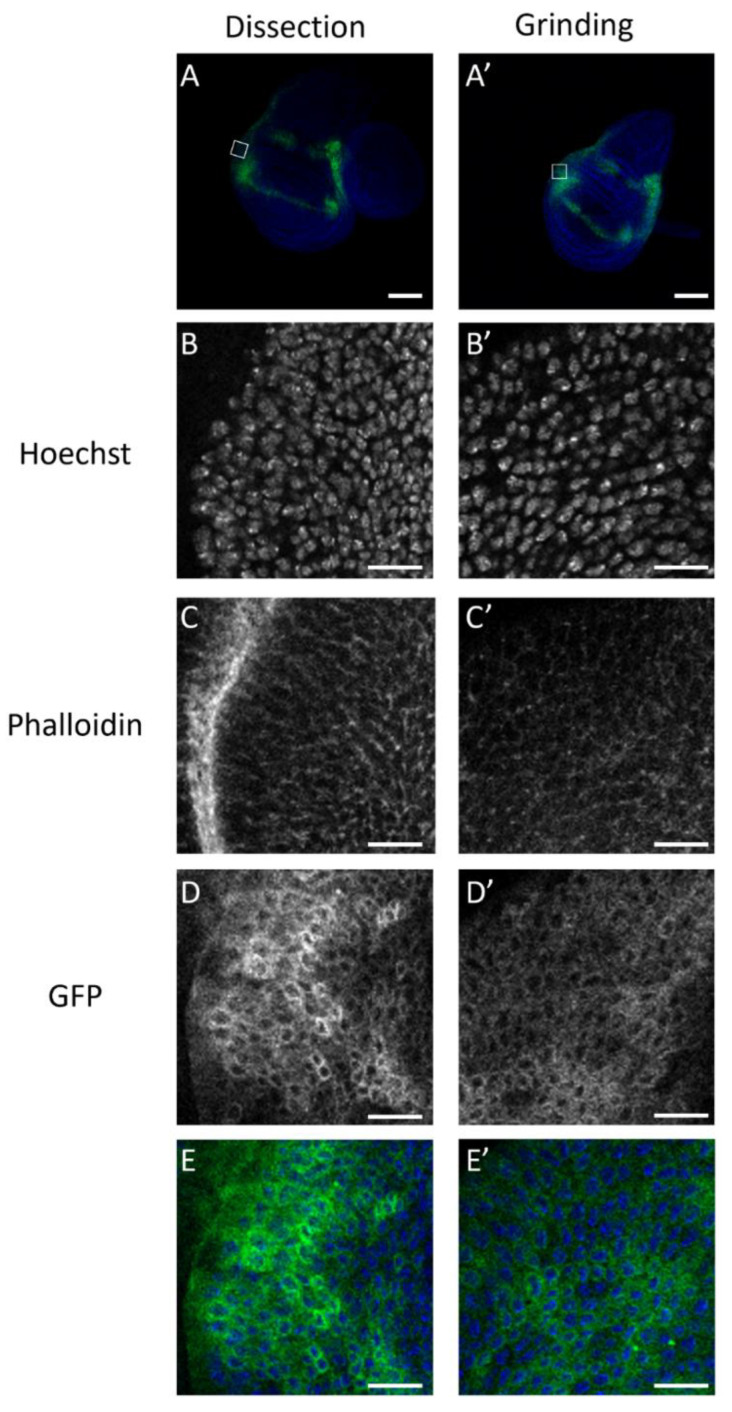
Cell structure in wing imaginal discs recovered by dissection or mass-enrichment protocol. Cell structure was assessed in wing imaginal discs recovered by dissection (**left** column) or mass-enrichment protocol (“grinding”, **right** column). Confocal images were acquired with 10X objective, and only a slice of this pseudocolumnar epithelium is shown (**A**,**A’**). Images (**B**–**E’**) correspond to the same wing imaginal discs and were acquired with a 63X objective. They show a zoomed-in view of the white boxes in (**A**,**A’**). Nuclei were visualized using Hoechst (**B**,**B’**), plasma membrane using phalloidin ATTO655 (**C**,**C’**). GFP is coupled to mCD8 protein, whose expression is driven by the *vg-GAL4* driver (**D**,**D’**). Representative overlays of nuclei staining (blue) and GFP (green) are shown in whole discs (**A**,**A’**) or in an enlargement of the wing imaginal disc posterior zone. Images E and E’ are an overlay of ((**B**,**D**,**B’**) and (**D’**)), respectively. Scale bars correspond to 100 µm (**A**,**A’**) or 10 µm (**B**–**E’**).

The tissue structure was also explored using fluorescent staining of *vg-GAL4*, mCD8-GFP, wing imaginal discs to stress the quality of the wing imaginal discs recovered by the mass enrichment protocol. Hoechst and phalloidin-ATTO 655 stained the nucleus and the plasma membrane, respectively. mCD8GFP protein is known to localize at the plasma membrane. The experiment was performed in parallel on wing discs isolated by dissection or mass enrichment protocol. The results in Figure 5 show that the wing disc cells obtained by the mass enrichment protocol have a normal structure. The results in Figure 5 show that the wing disc cells obtained by the mass enrichment protocol have a normal structure. The slight differences in intensity or morphology observed for staining within a single image (e.g., Figure 5C) or between images obtained under the two conditions (dissection or mass enrichment protocol) can be explained by the fact that the imaginal disc is a folded pseudo-columnar epithelium. Thus, the image plane is not at the same apical-basal level for all cells. The results do not reveal any significant difference in the quality of the discs recovered by both methods. 

### 3.2. Final Protocol (See Supplementary File S1 for Details)

**Figure 6:**
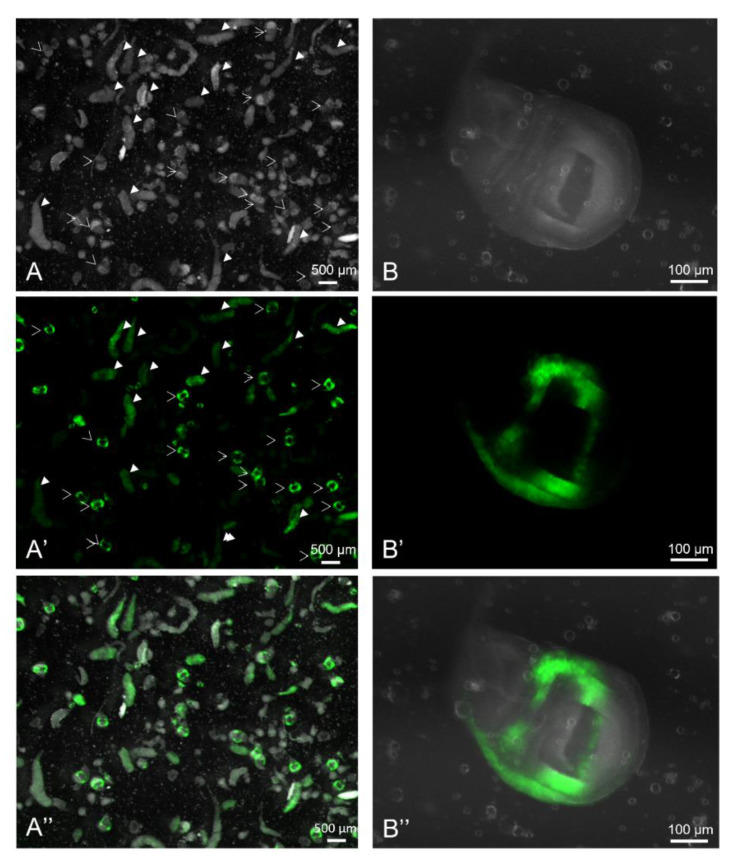
Yield and purity of the mass enrichment protocol. Overview of the resulting material obtained at the end of the procedure under white light (**A**,**B**) or GFP-exciting light (**A’**,**B’**). (**B**–**B’’**) show wing imaginal discs (the posterior compartment is on the bottom) corresponding to a 6.4 times magnification of (**A**–**A**’’). Empty arrowheads point to wing imaginal discs, and filled arrowheads point to salivary glands. Procedure (protocol E) was performed on larvae expressing GFP under the control of the *vestigial* driver.

Flushing the side of the tubes with water allows the recovery of L3 larvae from synchronized egg-laying. After rinsing in water to remove the growth medium, larvae are transferred in GentleMACS C tubes and ground in Ringer solution. The ground material is filtered through several strainers of decreasing meshes to separate organs by size; this mainly removes cuticles and fat bodies. These steps are repeated five times to ensure the good separation of organs without damaging them. The filtered organ suspension (100–200 µm) then undergoes a sedimentation step to remove gut pieces, fat body, and some other non-discs organs. Finally, a Ficoll density gradient allows the separation of imaginal discs from other organs. Salivary gland pieces constitute the primary contaminant (Figure 1 and Figure 6). Wing imaginal cells can be isolated and separated from salivary gland cells using a dissociation and a filtration step.

## 4. Discussion

The increasing use of global approaches to study gene expression or protein regulation justifies the development of mass purification techniques for *Drosophila* imaginal discs. By studying protocols from the 1960–1970s, we set up a protocol that allows recovery of third-instar larvae wing imaginal discs without manual dissection. To do so, we had to set up every step, from the grinding of larvae to the density gradient (Figure 2 and Supplementary Figure S1). During our protocol set-up, a compromise was made between purity and quantity. Therefore, the selected protocol allows obtaining fractions consisting of 50% of wing imaginal discs (Figure 2). There are two types of contaminants: salivary glands, the most abundant ones, and rare other imaginal discs (mainly eye-antenna discs). However, if pure fractions are needed, wing imaginal discs can be easily picked out with plyers. Moreover, if the objective is to recover the cellular fraction corresponding to the discs, a dissociation step followed by filtration allows removing the cells of the salivary glands.

Indeed, since they are much bigger than those of imaginal discs, passing the solution through a 40 µm strainer is enough to separate them from the wing imaginal disc cells of interest. The advantages and disadvantages of the protocol presented in this article are summarized in Supplementary Figures S4 and S5, which compare the pros and cons of some existing protocols. 

The protocol we selected has a yield of 11.5%, which is low compared to manual dissection yield, considered around 85%, by taking into account the organs that might be lost or damaged during the process. However, this mass enrichment protocol presents advantages over manual dissection.

(i) We used around 600 larvae as an input to obtain, on average, 140 wing imaginal discs. Depending on the researcher, this amount can be dissected in about two to three hours. However, if more wing imaginal discs are needed, the dissection time increases proportionally. One of the advantages of the mass enrichment protocol is that it can process several samples simultaneously by using parallel filtration montages and doing the filtration and centrifugations (sedimentation and Ficoll density gradient) at the same time (Figure 1). Therefore, its duration is only slightly impacted and should not take more than three hours.
(ii) When high amounts of organs are needed, multiple researchers usually dissect them together. As each researcher has their own method and ease of dissection, which may be influenced by the time spent dissecting, this may pose reproducibility problems. The enrichment protocol is standardized and can be performed entirely by one researcher, reducing reproducibility issues, which constitutes another advantage over manual dissection.
(iii) Since dissection allows only one sample to be processed at a time, this induces a delay in the treatment of each condition. Our protocol does not present this disadvantage, as several samples can be treated almost simultaneously. Thus, it allows a more homogenous treatment between conditions.

As mentioned in Section 3.1, this protocol requires upstream preparation: for all the individuals to be at the same stage, we recommend transferring the adults to fresh tubes every 24 h. As a reference, we obtain 2.5 mL of larvae (around 600 L3 larvae) with six vials of stable stock lines. When high amounts of material or numerous conditions are needed, these preparation steps can be facilitated using larger vials or even bottles. According to offspring viability, the number of necessary vials is up to the researcher’s decision. While the amount of work necessary to produce enough larvae seems large compared to that required for dissection, this technique allows the larvae to be harvested simultaneously, avoiding the treatment delay between each larva. Pooling all the larvae from a larger number of tubes also decreases the batch effect of each vial.

If larvae of interest are obtained by crossing and represent only 50% of the progeny, the number of vials must be doubled to obtain an equal amount of wing imaginal discs. Depending on the parents’ genotypes, only a fraction of the offspring may be of interest in certain conditions. Collecting these individuals based on their specific phenotypes (Tb, CyOGFP, etc.) is possible, but this would add a time-consuming step. In this case, using a fluorescent protein expressed in the population of interest presents two advantages. Firstly, it allows the identification of discs of interest at the end of the mass enrichment without selecting the larvae before starting the protocol. Secondly, when a large amount of disc is required to isolate cells of interest, the dissociation and filtration step allows obtaining wing imaginal cells that can be sorted by flow cytometry. Depending on the experimental design, other solutions can be considered, such as tagged proteins of interest and magnetic cell sorting [24]. 

Interestingly, this protocol can also be adapted to isolate other *Drosophila* organs by modifying the filtration or the Ficoll gradient steps using the indications detailed in this specific part. Moreover, to facilitate the tracking of organs throughout the gradient, we adapted a beta-galactosidase staining protocol without fixation to color the organs blue and have a visual clue of their localization [20] (Supplementary Figure S2). This staining can be applied to help isolate other organs.

Older protocols were able to graft the collected organs, which became normal adult structures [14]. We did not test this ability. However, by using a cell viability assay (Figure 3) and controlling the apoptosis level (Supplementary Figure S3), we showed that this protocol does not induce more damage than the dissection of wing imaginal discs. The mass enrichment protocol is standardized and achievable quickly, allowing a more homogenous treatment of wing imaginal discs. In the case of experiments that necessitate the analysis of a large number of imaginal discs, this constitutes a considerable advantage of a mass purification protocol. Remarkably, our analysis of cell viability strongly emphasizes the importance of cell buffer (Figure 3) and shows that cell survival is higher in Ringer buffer than in the PBS buffer, yet is commonly used for dissections.

As another quality control, we compared the expression of GFP in wing imaginal discs in which the vestigial driver drove GFP expression. Visual examination showed no notable difference in GFP levels between discs (see Figure 5 and Figure 6 for GFP-expressing wing imaginal discs recovered through the mass enrichment protocol). The percentage of GFP-positive cells estimated through flow cytometry corresponding to discs recovered by mass enrichment protocol and dissection is quite similar. The slight observed difference can be easily explained by the presence of a small number of other organs, such as eye-antennae discs, which “dilute” the GFP-positive cells when using a mass enrichment protocol. Nevertheless, GFP analysis shows that obtained discs are homogenous and that the purification protocol is reproducible. In many approaches, the cells of interest represent only a subpopulation of the cells in the imaginal disc. In this case, obtaining a sufficient number of cells is difficult to achieve by manual dissection. One of the interests of this approach is to allow a large quantity of a subpopulation of imaginal disc cells to be rapidly obtained by coupling the mass enrichment protocol to cell sorting by flow cytometry.

## 5. Conclusions

In summary, this paper shows that this mass enrichment protocol allows the rapid purification of a large quantity of wing imaginal discs of equivalent quality to manually dissected discs for multiple samples simultaneously and by a single investigator. Wing imaginal discs recovered by mass enrichment allow the isolation of wing imaginal disc cells suitable for numerous applications such as cytometry analyses (Figure 3 and Figure 4), transcriptomics, and proteomics.

## Data Availability

The data that support the findings of this study are available from the corresponding authors, I.G. and J.C., upon reasonable request.

## Supplementary Materials

The following supporting information can be downloaded at https://www.mdpi.com/article/10.3390/biology11101384/s1, Figure S1: Overview of the GentleMACS^TM^ programs tested; Figure S2: Example of a Ficoll gradient with β-galactosidase-stained wing imaginal discs; Figure S3: Apoptosis staining in the wing discs according to the disc enrichment method; Figure S4: Comparison of wing imaginal discs isolation protocols; Figure S5: Summary table of pros and cons of available imaginal discs isolation protocols; File S1: Mass enrichment protocol.
