# Supplementary material for: Mass Purification Protocol for Drosophila melanogaster Wing Imaginal Discs: An Alternative to Dissection to Obtain Large Numbers of Disc Cells
Source: Biology (Basel). 2022 Sep 22;11(10):1384. doi: 10.3390/biology11101384 (PMC9598552; doi:10.3390/biology11101384)
Supplement: Supplementary file 1 [file biology-11-01384-s001.zip › Fig_supp_Hoareau et al_cor_submited proof.pdf]

Test		Larvae disrupted?	Intact discs?
M	« liver » (1 or 2)	-	NT
M	« liver » (1 or 2) + « brain » (1)	+	-
M	« brain » (2)	++	-
C	« liver » (2)	-	NT
C	« liver » (1) + « brain » (2)	+	-
C	« brain » (2)	++	++

Liver: 1(48 rpr, 15 s) or 2(78 rpr, 24 s) / Brain: 1(116 rpr, 36 s) or 2(100 rpr, 30 s)

Supplementary Figure S1: Overview of the GentleMACS^{TM} programs tested. The GentleMACS^{TM} (Miltenyi) device can be used with either C or M kinds of tubes and display various pre-registered programs. We tested several combinations to identify conditions efficient enough to open larvae and detach discs without damaging these organs. NT : not tested, ++ : successful and optimal try, + : successful but not optimal try, - : unsuccessful try

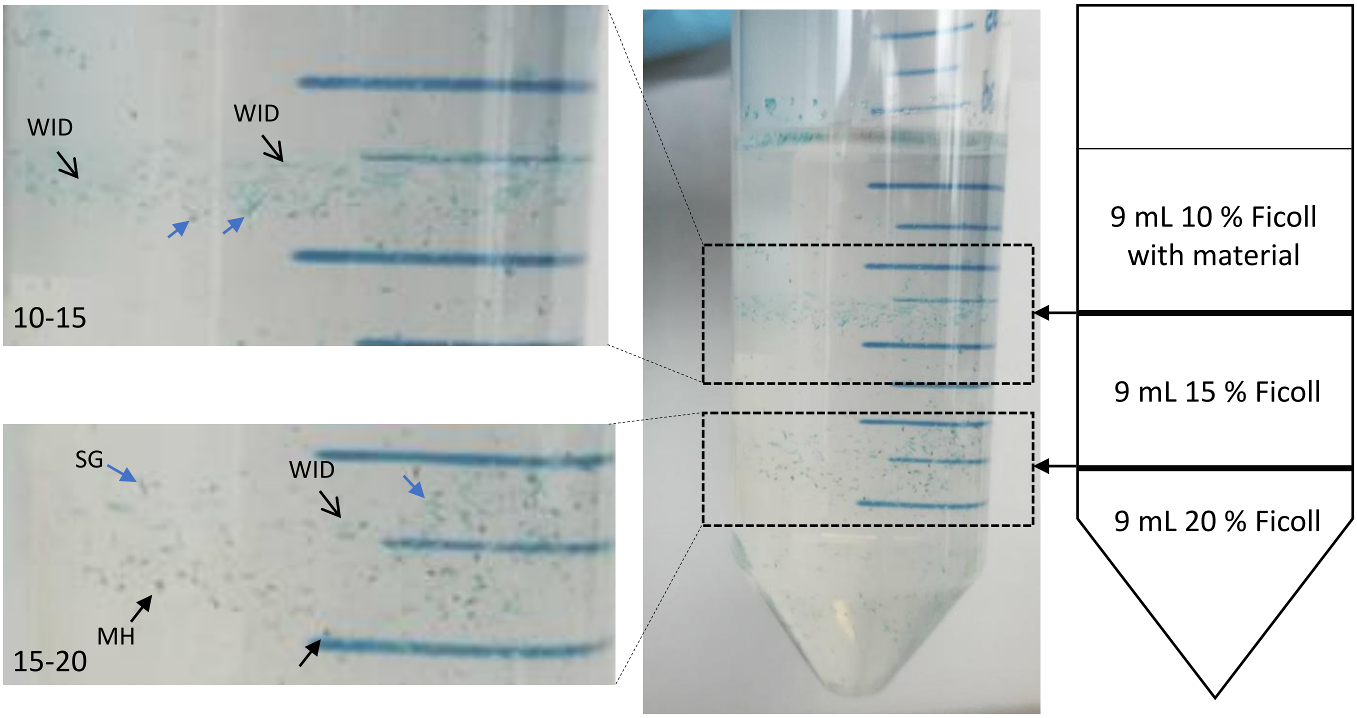

Supplementary Figure S2: Example of a Ficoll gradient with β-galactosidase-stained wing imaginal discs. Ficoll gradient after migration with β-galactosidase-stained wing imaginal discs. Inserts show magnification of the 10-15 and 15-20 interphases, blue arrows point to salivary glands (SG), and black arrows point to mouth hooks (MH). Smaller blue structures are wing imaginal discs (WID).

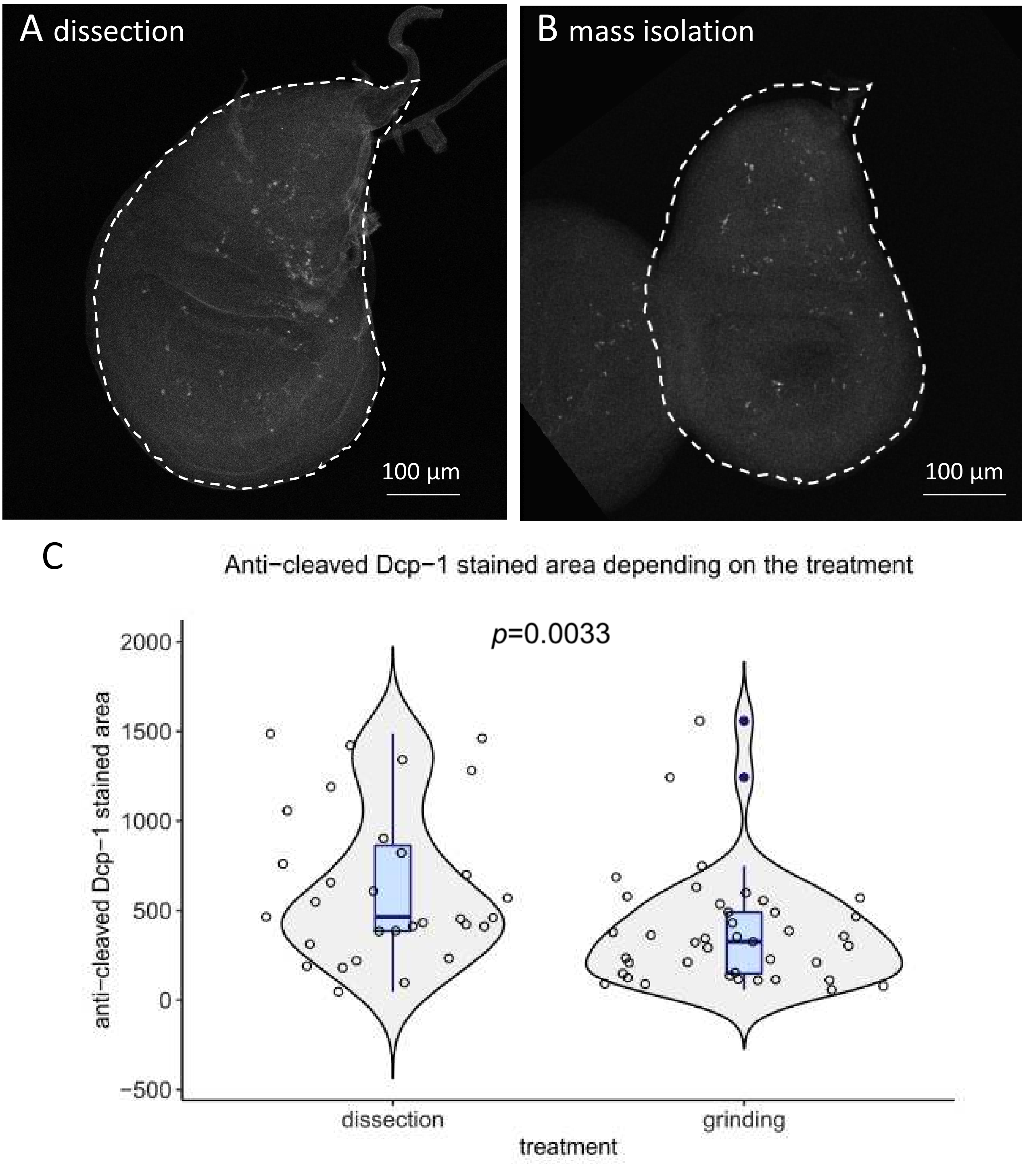

Supplementary Figure S3: Apoptosis staining in the wing discs according to the disc enrichment method.

Protocol	Buffer	Grinding method	Filtration	Ficoll gradient	Yield	Purity	Quality
Fristrom and Mitchell, 1965	Ringer	Meat grinder	1.43 mm, 0.5 mm, n°5 silk sifting cloth	1st gradient 14:19%, 3 min 450 xg (estimated speed*) 2nd 14% layer on top of linear gradient (14 to 24)	10 to 20%	95% containing leg, wing and eye-antenna imaginal discs	Transplantable
Zweidler and Cohen, 1971	Organ medium (5 mm disodium glycerophosphate, 10 mm KH2PO4, 30 mm KCl, 10 MM MgC12, 3 rum CaC12, 162 RIM sucrose pH 6 .8)	Hand made automatic dissection device	Nylon net	Linear gradient 2 to 5%, no centrifugation but homemade sedimentation device	Unclear (« From 500 g of larvae, about 0.4 g of imaginal discs are obtained. »)	Unclear (« Contamination, as estimated by microscope observation, is about 20% by mass »)	Unclear, mentions that the technique has been used to study nuclear proteins and nuclear acids
Marty *et al.*, 2014	PBS	GentleMACS™ program m_liver_01_01	500 µm	16:25, 1h 4332 xg (estimated speed*)	1000 to 1500 wing discs for 5 mL of larvae. According to our calculations : 42 to 62%	Low after Ficoll gradient (enriched in wing imaginal discs) but sorted with Biosorter afterwards	ChIP-on-chip done (see Schertel *et al.*, 2015)
Hoareau *et al.*, 2022	Ringer	GentleMACS™ program m_brain_02_01	500/300/200 µm + 100 µm	10:15:20:25%, 20 min 80 xg	11.5%	50% directly after gradient due to salivary glands, but easy removal	Normal GFP expression and death rate

Supplementary Figure S4: Comparison of wing imaginal discs isolation protocols.

Protocol	Pros	Cons
Fristrom and Mitchell, 1965	Very pure<br>Different kind of imaginal discs<br>Good quality	Different kind of imaginal discs<br>Meat grinder not available<br>2 Ficoll gradient including one continous
Zweidler and Cohen, 1971	No crushing (automated dissection-like device with needles)	Lots of homemade devices difficult to reproduce<br>Unclear on yield and purity
Marty *et al.*, 2014	High amount of discs<br>Minimalist protocol	Use of PBS<br>Need of a BioSorter
Hoareau *et al.*, 2022	Pretty high amount of discs<br>Possibility to treat simultaneous samples<br>Pure cells after dissociation and filtration<br>Good quality	Yield<br>50 % purity right after gradient

Supplementary Figure S5: Summary table of pros and cons of available imaginal discs isolation protocols.
