# Supplementary material for: Mass Purification Protocol for Drosophila melanogaster Wing Imaginal Discs: An Alternative to Dissection to Obtain Large Numbers of Disc Cells
Source: Biology (Basel). 2022 Sep 22;11(10):1384. doi: 10.3390/biology11101384 (PMC9598552; doi:10.3390/biology11101384)
Supplement: Supplementary file 1 [file biology-11-01384-s001.zip › Hoareau et al_Sup File 1_submited_31 08 22.pdf]

Materials and Reagents

A. Larvae recovery and grinding
    1. *Drosophila* third instar larvae in their rearing tubes
    2. Water
    3. 1X Ringer (3 mM NaCl, 182 mM KCl, 46 mM $CaCl_2$)
    4. GentleMACS™ C tubes (Miltenyi, 130-093-237)

B. Filtration, sedimentation, and gradient
    1. 50 mL polypropylene tubes (Corning-Falcon, 352070)
    2. 25 mL pipettes (Corning-Falcon, 356535)
    3. 10 mL pipettes (Corning-Falcon, 357551)
    4. 1X Ringer (3 mM NaCl, 182 mM KCl, 46 mM $CaCl_2$)
    5. pluriStrainers 500 µm, 300 µm, 200 µm, 100 µm (PluriSelect, 43-50500, 43-50300, 43-50200, 43-50100)
    6. Funnel (PluriSelect, 42-50000)
    7. Ficoll PM400 30% (w/v), 25% (w/v), 20% (w/v), 15% (w/v) solutions prepared in 1X Ringer solution from (Thermofisher 11590724)

C. Rehydration and pelleting
    1. 10 mL sterile pipettes (Corning-Falcon, 357551)
    2. 1X Ringer (3 mM NaCl, 182 mM KCl, 46 mM $CaCl_2$)
    3. SmartStrainers 70 µm (Miltenyi, 130-098-462)
    4. Sterile filter tips 1000 µL (Starlab, S1126-7810)
    5. 1.5 mL tubes (Eppendorf, 0030120086)

D. Dissociation and filtration
    1. 1.5 mL tubes (Eppendorf, 0030120086)
    2. 1X Ringer (3 mM NaCl, 182 mM KCl, 46 mM $CaCl_2$)
    3. Sterile filter tips 1000 µL (Starlab, S1126-7810)
    4. Protease 0.1% solution diluted from in 1X Ringer from (Sigma, p8811))
    5. PluriStrainer Mini 40 µm (PluriSelect 43-10040)

Equipment

1. Wash bottles
2. gentleMACS™ Dissociator (Miltenyi)
3. Calibration checked pipettes 20-200 µL, 100-1000 µL
4. Eppendorf 5810R refrigerated centrifuge with swing bucket rotor (A-4-62) or equivalent with settings for acceleration and deceleration
5. Eppendorf ThermoMixer® or other heating and shaking device

Procedure

A. Larvae collection and grinding
    1. Flush the sides of the tubes with a wash bottle filled with water to collect larvae in an empty beaker.
       Note. To ensure that all larvae are third instar, transfer the parents to a fresh medium every 24 hours and take care not to overcrowd the tubes. See Note 1 for the number of larvae needed.
    2. Rinse the larvae with water to get rid of the remaining medium residues.
    3. Transfer the larvae to a 500 µm strainer to eliminate the water.
    4. Transfer the larvae to a GentleMACS C tube containing around 2.5 mL of 1X Ringer.

Note. For optimal grinding, do not put over the 2.5 mL graduation of larvae per tube, otherwise, all larvae may not be correctly ground without adding grinding cycles and therefore, adding time to the duration of the protocol.

5. Complete the tube with 1X Ringer up to the 10 mL graduation.
6. Grind larvae using the “brain_02_02” program of the GentleMACS™. (100 rpm, 30 sec)
   Note. Centrifuge quickly the C tube to pellet the larvae debris that could be stuck in the cap helix

B. Filtration, sedimentation, and gradient

1. Filter the ground material through an assembly of 500, 300, and 200 µm strainers (see Fig. 1) and recover the filtrate in a 50 mL tube (named “1”, which contains the imaginal discs released).
   Note. Flush the helix with a wash bottle filled with 1X Ringer to be sure to recover most material.
2. Put back in the C tube what did not pass the 500 µm sieve and add 1X Ringer up to 10 mL and repeat steps A6-B1 until all larvae are properly disrupted (usually 5 cycles are sufficient)

   Filtrate the content of the “1” tube through a 100 µm strainer and recover what is retained in the strainer (size>100 µm) in a fresh 50 mL conical tube named “2” by turning it upside down on top of the tube *Note. In the first round of filtration, the liquid content that passes 100 µM is rich in lipids. It can be further filtered through a 30 µm strainer to get a solution very useful for pipette coating thus avoiding the organs to stick to the pipettes during downstream steps.*

   Note. If the “2” tube is full, pass its content through the 100 µm filter to discard some liquid.
3. Centrifuge the “2” tube (containing the 100-200 µm material) at 200 *xg* 1 min 4°C.
4. Discard the supernatant and resuspend the pellet in 20 mL of 1X Ringer.
5. Centrifuge at 50 *xg* 1 min 4°C.
6. Discard the supernatant and resuspend the pellet in 20 mL of 1X Ringer.
7. Centrifuge at 10 *xg* 1 min 4°C.
8. Wait for 20 seconds.
9. Discard the supernatant and resuspend the pellet in 20 mL of 1X Ringer.
10. Repeat steps B7-B8
11. Discard the supernatant and resuspend the pellet in 7.5 mL of 1X Ringer.
12. Add 2.5 mL 30 % Ficoll (w/v) for a 7.5 % Ficoll final concentration.
13. Mix well and centrifuge at 50 *xg* 5 min 4°C (acc. 9, dec. 3)
14. Discard the supernatant and resuspend the pellet in 4 mL 1X Ringer.
15. Add 2 mL Ficoll 30% (w/v) for 10 % Ficoll final concentration and mix well. Store at +4°C while preparing the gradient.
    Note. If there is a lot of material, increase the volume of the 10 % layer (Ringer + Ficoll) otherwise the layer will be too heavy for the gradient and will go under the 15 % layer.
16. In a new 50 mL tube, add 9 mL of 25 % Ficoll (w/v).
17. Centrifuge 3220 *xg*, 3 min, 4°C (acc. 3, decc. 1)
18. Carefully pour the 20% (w/v) layer (14 mL) on top of the 25% (w/v) layer.
19. Carefully pour the 15% (w/v) layer (14 mL) on top of the 20% (w/v) layer.
20. Coat the pipette with the solution kept in step B2.
21. Carefully pour the layer containing the material (from step B15).
22. Centrifuge 80 *xg*, 20 min, 4°C (acc. 3, decc. 1)

C. Rehydration and pelleting

1. Coat the pipette with the solution kept in step B2.
2. Carefully pipet the interface between the 15 and 20% (w/v) layers (2nd and 3rd layers).
3. Collect the interface in a 70 µm strainer.
4. Rinse with 1X Ringer.
5. Place the strainer with the material in a box containing a large volume of 1X Ringer (about 20 mL, depending on the size of the box) so that the material in the strainer is fully covered with buffer.
6. Leave the material to rehydrate for at least 20 min at RT.

   Note: you can check at this point how many wing imaginal discs you recovered with a stereomicroscope.

7. Transfer the material to a 1.5 mL microtube.

   Note. To avoid losing material sticking to the tips, flush the material out of the strainer with some Ringer into the microtube rather than pipetting it.

8. Centrifuge 4000 *xg*, 3 min, 4°C: wing imaginal discs are in the pellet.

   Note: Stop here if you need undissociated wing imaginal discs.

D. Dissociation

1. Discard supernatant from the C8 step.
2. Resuspend the pellet in 1 mL of 0,1%.protease (w/v)
3. Incubate for 20 min at 25°C in a Thermomixer with gentle shaking (300 rpm).
4. Complete the dissociation with gentle ups and downs with a 1000 µL tip.
5. Centrifuge 4000 *xg*, 3 min, 4°C: wing imaginal disc cells are in the pellet.
6. Resuspend the pellet in Ringer 1X.
7. Filter the solution through a 40-µm filter: wing imaginal disc cells pass through, and salivary gland cells stay in the filter.

Note 1: It takes around 600 larvae (= 2,5 mL) to get 100 to 150 wing imaginal discs.
